# Computed tomographic pulmonary angiographic findings to predict adverse outcomes in acute pulmonary embolism

**DOI:** 10.1186/cc14407

**Published:** 2015-03-16

**Authors:** P Tajarernmuang, J Euathrongchit, C Liwsrisakun, A Deesomchok, T Theerakittikul, C Bumroongkit, C Pothirat, A Limsukon

**Affiliations:** 1 Chiang Mai University, Chiang Mai, Thailand

## Introduction

Computed tomographic pulmonary angiography (CTPA) has been used as a standard tool for diagnosing an acute pulmonary embolism (APE). The right ventricular (RV) strain signs may be used to predict adverse outcomes. However, the results are still controversial. The primary objective of our study was to evaluate the relationship between the RV strain signs and respiratory failure requiring mechanical ventilation or death in APE. The secondary objective was to identify clinical factors which related to those outcomes.

## Methods

CTPA and the medical records of patients with suspected APE on admission from June 2011 to March 2013 were reviewed. RV dysfunction signs included right ventricular to left ventricular (RV/LV) diameter ratio, interventricular septal shift, main pulmonary artery to ascending aorta (mPA/AA) diameter ratio, IVC contrast reflux, SVC diameter, IVC diameter, PA diameter and azygos vein diameter. Clinical factors included cardiovascular, respiratory parameter and also time to diagnosis and treatment.

## Results

There were total of 36 cases with suspected APE on admission. Ten patients required mechanical ventilation (27.8%) and seven patients died (19.4%). Interventricular septal (IVS) shift was a significant risk factor of in-hospital death (85.7% vs. 27.6%, *P *= 0.008) and respiratory failure (70% vs. 26.9%, *P *= 0.026). The sensitivity, specificity, positive predictive and negative predictive values of IVS shift to predict in-hospital death were 85.7%, 70%, 42.8% and 95.5%, respectively. The sensitivity, specificity, positive predictive and negative predictive values of IVS shift to predict respiratory failure were 70%, 73.1%, 50% and 86.4%, respectively. The ratios of RV to LV diameter and the ratio of main pulmonary artery to ascending aorta diameter tended to be higher in the nonsurvivor group. The clinical factor that predicted mortality was the PaO_2_ to FiO_2_ ratio (P/F ratio). Mean P/F ratio in survivor and nonsurvivor groups was 246.1 ± 94.1 vs. 132.2 ± 78.1, respectively (*P *= 0.011). P/F ratio ≤150 was the best predictor of mortality (66.7% vs. 8.7%, *P *= 0.008).

## Conclusion

The IVS shifting from CTPA and P/F ratio ≤150 helps predict poor outcomes in APE.
